# Expression of Inflammatory-Related NFκB Genes in Iranian Patients with Pterygium: A Case-Control Study

**DOI:** 10.22088/IJMCM.BUMS.7.3.169

**Published:** 2018-10-22

**Authors:** Seyed Mohammad Salar Zaheryani, Mohammad Essmail Ebrahimi, Abdollah Kasaei, Amir Roointan, Mahmood Nejabat, Mehdi Dianatpour, Meisam Meisam, Mohammad Reza Talebnejad, Fakhraddin Naghibalhossaini

**Affiliations:** 1 *Poostchi Ophthalmology Research Center, Shiraz University of Medical Sciences, Shiraz, Iran.*; 2 *Stem Cells Technology Research Center, Shiraz University of Medical Sciences, Shiraz, Iran.*; 3 *Department of Medical Biotechnology, School of Advanced Medical Sciences and Technologies, Shiraz University of Medical Sciences, Shiraz, Iran.*; 4 *Biochemistry Department, Shiraz University of Medical Sciences, Shiraz, Iran.*

**Keywords:** Pterygium, inflammation, gene expression, NF-kappa B, real-time RT-PCR

## Abstract

Pterygium is one of the most common eye conditions without any clear etiology. Some studies have suggested an association between sun exposure and pterygium, but others have proposed the role of genetic variations in its pathogenesis. To date, no study has investigated the association of inflammatory transcription factor, *NFκB* genes with pterygium in the Middle East. We examined the changes in expression of 3 inflammatory related *NFκB1, NFκB2*, and *RELA* genes in patients with pterygium. Thirty patients with pterygium and 30 age and sex-matched controls were enrolled in this case-control study. None of the participants showed any clinical signs of inflammation in their conjunctiva. Demographic information was obtained and the expression levels of three genes including *NFκB1*, *NFκB2*, and *RELA* were measured in their conjunctiva by real-time RT-PCR using gene-specific primers. Mean expression level of *NFκB1*, *NFκB2* and *RELA* genes in patients were 2.4±0.3, 1.9± 0.5, and 1.8±0.4 times higher than normal subjects, respectively. Higher levels of gene expression were observed in individuals with more outdoor activity and sun exposure. Moreover, a significant correlation was observed between the expression levels of *NFκB2* and *RELA* genes, suggesting a possible NFκB2- RELA heterodimer formation in patients with pterygium. This study has indicated a significant association between expressions of inflammatory-related *NFκB1*, *NFκB2* and *RELA* genes, and pterygium. Further studies to verify the role of inflammation in the pathogenesis of pterygium, may provide new targets for managing pterygia.

Pterygium is a common ocular disorder characterized by the growth of a wing-like connective tissue on the cornea (1) that can cause irritation, foreign body sensation, and conjunctival hyperemia. It also induces astigmatism and affects visual quality (1, 2). Pterygium occurrence is generally high in semi-dry regions, and its prevalence was reported to be 2% (3) while according to a cohort study in Iran the prevalence of pterygium in the urban population was 9.4% and 2.9% in one and both eyes, respectively (4).

Exposure to sunlight and genetic factors are the two main proposed contributors in the initiation and development of pterygium (5-7). Sunlight can induce inflammation, DNA damage, and secretion of growth factors, resulting in pterygium (8). So far, several reports have proposed the role of inflammation in pathogenesis, and post-surgery outcomes in pterygia (9-11). The nuclear factor kappa-light- chain- enhancer of activated B (*NF-κB*) family members including *NF-κB1* (*p50*), *NF-κB2* (*p52*), *RELA* (*p65*), *RELB* and *C-REL*, are the main mediators in initiating inflammation by inducing pro-inflammatory agents expression (12). Formation and activation of different dimers of *NF-κB* members mediate specific target gene expression in response to various stimuli (13). Various biological processes such as proliferation, apoptosis, differentiation, and migration are regulated through the *NF-κB *pathway with expression of more than 150 genes (14). Of the important relevant genes in pterygium are epithelial neutrophil activating peptide 78 (*CXCL5*), monocyte chemotactic protein 1 (*CCL2*), and matrix metalloproteinases (*MMPs*) (15). A few studies suggested the role of *NF-κB *in cellular responses to UV damage and hyperosmotic stress (16, 17) that might have a role in pterygium.

Siak et al., for the first time reported *NF-κB *pathway activation in pterygium in Singaporean patients (18). Another study at 2017 showed elevated levels of local pro-inflammatory cytokine and nitric oxide responses in pterygium of Algerian patients (19). Recently, a few studies have concluded that despite the role of *NF-κB* family members as an initiator of inflammation, their inhibition after the initial inflammatory insult, may prolong the process of inflammation and delay tissue repair (20, 21).

In this study, we evaluated the probable associations between the mean expression levels of *NFκB1*, *NFκB2* and *RELA* genes in the pterygium. Amongst all family members, these three proteins are more prevalent in activated form of NFκB heterodimers (12, 22). Dysregulation or aberrant expression of NFκB proteins were shown to be linked with neoplasms, autoimmune and inflammatory disorders (23-26). Therefore, the aim of this study was to examine the expression of these inflammatory-related genes in patients with pterygia in a case-control study.

## Material and methods

**Study subjects**

Specimens from 30 patients with pterygium and 30 controls were collected from Khalili Hospital, Shiraz, Iran between 2016 and 2017. Inclusion criteria were no history of prior ocular surgery and taking topical ocular medications other than lubricants. All patients with history of using anti-glaucoma medications, inflamed pterygium or pseudopterygium were excluded. Inflamed conjunctiva was considered as hyperemia, membrane, psudomembrane, and, follicular or papillary reaction. A true pterygium was considered if it had edges that could be elevated with forceps or under which a probe could be passed and usually arose from a pinguecula. The control subject's specimen consisted of 2×2 mm supranasal conjunctiva, taken from the patients who had undergone cataract extraction, at the end of surgery. The participants were examined before surgery and the controls had no ocular surface disease, pterygium or pinguecula. They were asked about their occupation and amount of sun exposure. Written informed consent was obtained from all participants. The study was approved by the local school of medicine research ethics committee, Shiraz University of Medical Sciences.

**RNA isolation and real time PCR quantification of** ***NFκB1*** **,** ***NFκB2*** **and** ***RELA*** **expression**

RNeasy Plus Mini Kit (QIAGEN, Netherlands) was used to isolate total RNA from case and control groups. From each sample, 1 µg of total RNA was converted into cDNA (final volume 20 µl) using QuantiTect reverse transcription kit (QIAGEN, Netherlands). Quantitative real time PCR of the synthesized cDNA molecules, was performed using AB15700 sequence detection system (Applied Biosystems, USA). *GAPDH* was used as the internal control gene. All experimental steps were performed based on the manufacturer is instructions. For real time PCR amplification, 1 μl of cDNA, 10 pmol of each specific primer (1 μl of each primer), 10 µl of 2× SYBR Green master mix, were mixed in a 20 µl final reaction volume. For each sample, the amplification was performed three times. We calculate the mean ± SD for each group,the individual data points, using 2^-ΔCT^ as previously described (27). Primers and their sequences are shown in table 1.

**Table 1:** Primer sequences for studied genes

Gene	Primer sequences (5′ → 3′)	Annealing temperature (°C)	Amplicon size (bp)
*NFκB1*	F: CTATGACCTGGATGACTCTT R: ATGTCTCCTTGTGCTAGTAA	60	165
*NFκB2*	F: CCAGTGATGGCTCCTT R: AACCTCAATGTCATCTTTCTG	60°C	182
*RELA* *GAPDH*	F: CTGCCAGATACAGACGAT R: GGGTCCGCTGAAAGG F: CCTAGATTATTCTCTGATTTGGT R: ATGTAGTTGAGGTCAATGAAG	60 60	99 115

**Table 2:** Demographic information of studied subjects

Parameters	Age (Years ±SD)	Males N(%)	Females N(%)	Outdoor occupation N(%)	Sun exposure (hr/day)
Cases	53 ± 3.8	15 (50%)	15 (50%)	25 (83%)	11 ± 2
Controls	55 ± 1.9	18 (60%)	12 (40%)	15 (50%)	6.5 ± 1.5

**Table 3:** Mean expression of NFκB1, NFκB2 and RELA

Gene	Mean ± SD (Case)	Mean ± SD (Control)	P value
*NFκB1*	0.22 ± 0.13	0.10 ± 0.06	P˂0.0001
*NFκB2*	0.18 ± 0.15	0.08 ± 0.06	P˂0.0001
*RELA*	0.25 ± 0.19	0.13 ± 0.10	P˂0.0001

**Fig. 1:**
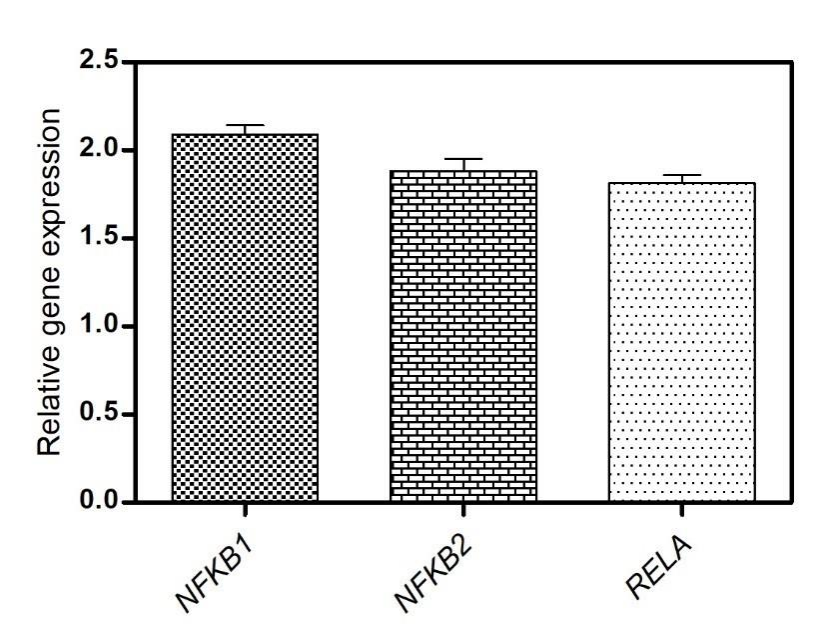
Relative genes expression in patients versus controls

**Statistical analysis**

Qualitative data were described in number (percent) and quantitative variables were expressed as the mean values ± SD. Normality of the data was checked by Kolmogorov-Smirnov test. Data of mean gene expression were analyzed by student t-test. Mean sun exposure was analyzed by student t-test. The correlations between expression levels of genes were assessed by Pearson’s correlation test. P value ˂0.01 was considered to be statistically significant.

## Results

The pterygium patients were 15 males and 15 females with mean age of 53, and the control group consisted of 18 males and 12 females with mean age of 55 years. Table 2 shows the demographic information of the subjects. Outdoor occupations included agriculture, construction, gardening, and fishing. The amount of sun exposure was stated by the participants. Study groups were matched for age and gender. Outdoor occupation rate was analyzed by chi-square test, which was statistically significant between the two groups (P= 0.003). Mean sun exposure between the two groups was evaluated by student t-test and was statistically significant (P= 0.002).

The relative genes' expressions in patients versus controls are shown in figure 1. Quantification of *NFκB1*, *NFκB2* and *RELA *expression showed a significant difference between cases and controls (P˂ 0.0001) (Table 3).

Moreover, a significant correlation was found between *NFκB2* and *RELA *expressions in patients as verified by Pearson’s correlation test (2-tailed). No statistically significant correlation was found between expressions of *NFκB2* and *NFκB1* or *RELA* and *NFκB1*.

## Discussion

A few studies have suggested that pterygium should be classified as a degenerative process (28), while others consider it as an unusual growth disorder (29, 30), or a dysfunction in limbal stem cells (31).

The results of epidemiologic experiments revealed some associations between pterygium with other sun-related eye disorders, such as cataract, basal cell carcinoma, and spheroidal degeneration (31-34). In vitro experiments showed the major role of ultra violet ray in initiating pterygium (35-37). Higher levels of vitamin D and higher occurrence of pterygium in men with more outdoor activities can be a confirmation for the indisputable role of sun exposure in the pathogenesis of pterygium (38); which are consistent with the results of our study.

Some reports proposed genetic susceptibility to pterygium. For instance, polymorphisms in genes of DNA repair system such as *Ku70* in non-homologous end-joining repair system (39) and human 8-oxoguanine glycosylase I (*hOGG1*) (40) can be considered as a genetic factor of predisposition to pterygium. Sometimes, polymorphisms in DNA repair system elements can predisposition to pterygium. Sometimes, polymorphisms in DNA repair system elements can be positive. For example, polymorphism of X-ray repair cross complementary 1 (*XRCC1*) gene might lead to decreased chance of pterygium development (41). In a study, increased levels of transforming growth factor (*TGF*-*β1*) expression in pterygium tissue of different atopic cases suggested the associations between this growth factor and pathogenesis of pterygium (42).

In this study, we attempted to find the role of inflammation in this disease. Hence, we evaluated the expression levels of three main genes of *NFκB* family including *NFκB1*, *NFκB2* and *RELA* in patients with pterygium and controls. Selecting *NFκB* family was due to their central role as an initiator of different inflammation pathways (12). Our results showed significant differences in expression levels of three *NFκB* family genes among the participants. Mean expression levels of *NFκB1*, *NFκB2* and *RELA* genes were 2.4±0.3, 1.9± 0.5, and 1.8±0.4 times higher in patients with pterygium in comparison with controls, respectively. These findings are consistent with the finding reported by Siak et al (18). A study by Torres et al., 2011, on 21 cases and 13 controls revealed that patients with pterygium had alterations in *NFκB* pathways (43), which is in line with our findings. They also showed higher levels of *NFκB* expression in ipsilateral pterygium-free conjunctiva. Investigating the other eye of the unilateral pterygium patients and following them to see any pterygium development can clarify the causative role of *NFκB* expression in pterygium that can be targeted pharmacologically; by retinoic acid, for example.

As noted by Siak et al., (18), Torres et al., (43) and also in this study, participants with clinical inflammation were excluded from the study. This signifies the presence of subclinical inflammation.

Further analysis of demographic information of patients indicated a significant correlation between outdoor activity and *NFκB* family expression levels. Most of the individuals with pterygium had more outdoor activities and sun exposure in comparison with the controls, suggesting the correlation between sun exposure, inflammation and pathogenesis of pterygium.

However, more studies are needed to verify the role of inflammation in the etiology of pterygium.

We also found a positive correlation between *NFκB2* and *RELA* genes in the pterygium patients. These two proteins can form NFκB heterodimer. Activated forms of NFκB can either contain NFκB1 and RELA or NFκB2 and RELA proteins. A heterodimer including NFκB2 and RELA contains the necessary transactivation domains that can lead to gene induction and initiation of inflammation cascades. Further investigations are required to confirm the NFκB heterodimer formation and its clinical importance in the etiology of pterygium.

In conclusion, in this study, by measuring *NFκB* family expression levels, as the key players in inflammatory conditions, we provided evidence for a possible association between inflammation and pterygium and found generalizability of this expression in Iranian population. We also showed a positive correlation between sun exposure and expression of inflammatory elements. This study showed a correlation between inflammation through *NFκB* family members and pterygium that indicates a subclinical inflammation in pterygium that might be a target for therapy. In other words, even in uninflamed pterygia, anti-inflammatory drugs might stop the progression of the disease. Further investigations including measuring NFκB protein levels and other markers of inflammations are recommended.
